# Layer-By-Layer Fabrication of Thicker and Larger Human Cardiac Muscle Patches for Cardiac Repair in Mice

**DOI:** 10.3389/fcvm.2021.800667

**Published:** 2022-01-06

**Authors:** Lu Wang, Jianyi Zhang

**Affiliations:** ^1^Department of Biomedical Engineering, School of Medicine, School of Engineering, University of Alabama at Birmingham, Birmingham, AL, United States; ^2^Division of Cardiovascular Disease, Department of Medicine, School of Medicine, University of Alabama at Birmingham, Birmingham, AL, United States

**Keywords:** myocardium, myocardial infarct, heart failure, tissue engineering, pluripotent stem cells

## Abstract

The engineered myocardial tissues produced *via* most manufacturing techniques are typically just a few dozen micrometers thick, which is too thin for therapeutic applications in patients. Here, we used a modified layer-by-layer (LBL) fabrication protocol to generate thick human cardiac muscle patches (hCMPs) with thicknesses of ~3.75 mm. The LBL-hCMPs were composed of a layer of endothelial cells (ECs) sandwiched between two layers of cardiomyocytes (CMs): both cell populations were differentiated from the same human induced pluripotent stem cell line (hiPSCs) and suspended in a fibrin matrix, and the individual layers were sutured together, leaving channels that allowed the culture medium to access the internal cell layer. The LBL-hCMPs were cultured on a dynamic culture platform with electrical stimulation, and when compared to Control-hCMPs consisting of the same total number of hiPSC-ECs and -CMs suspended in a single layer of fibrin, hiPSC-CMs in the LBL-hCMPs were qualitatively more mature with significantly longer sarcomeres and expressed significantly higher levels of mRNA transcripts for proteins that participate in cardiomyocyte contractile activity and calcium handing. Apoptotic cells were also less common in LBL- than in Control-hCMPs. The thickness of fabricated LBL-hCMP gradually decreased to 0.8 mm by day 28 in dynamic culture. When the hCMP constructs were compared in a mouse model of myocardial infarction, the LBL-hCMPs were associated with significantly better measurements of engraftment, cardiac function, infarct size, hypertrophy, and vascularity. Collectively these observations indicate that our modified LBL fabrication protocol produced thicker hCMPs with no decline in cell viability, and that LBL-hCMPs were more potent than Control-hCMPs for promoting myocardial repair in mice.

## Introduction

Less than 1% of cardiomyocytes in the hearts of adult humans are replaced each year (1), and this meager capacity for self-renewal is far too low to regenerate the myocardial tissue that is lost to cardiac injury or disease. Thus, the damage caused by a myocardial infarction (MI) typically leads to adverse cardiac remodeling, hypertrophy, contractile dysfunction, and heart failure (2). Adult cardiomyocytes are also essentially non-proliferative in culture, which severely limited the number of cells available for therapeutic applications and investigational studies, but this scarcity was alleviated by the development of human induced-pluripotent stem cells (hiPSCs), which can proliferate indefinitely and be differentiated into cells of any lineage, including cardiomyocytes (hiPSC-CMs) (3). Direct intramyocardial injections of hiPSC-CMs have improved recovery from MI in rodents, swine, and non-human primates (4), but the proportion of cells that remain engrafted at the site of administration is exceptionally low (5, 6). Engraftment rates are somewhat higher when the cells are incorporated into engineered human cardiac muscle patches (hCMPs), which are designed to replicate the structural and functional properties of native myocardial tissue with the greatest possible fidelity and, consequently, can also provide a valuable platform for *in-vitro* mechanistic studies, disease modeling, and drug testing (7, 8). The structural support provided by an implanted hCMP can also limit the progression of myocardial disease by preventing left ventricular (LV) dilation and over-stretch of cardiomyocytes located in the border zone of the infarct (9).

The Japanese health ministry has approved studies of hiPSC-derived tissues in a small number of patients (10), but the hCMPs produced *via* most manufacturing techniques are typically just a few hundred micrometers thick, which has impeded the translation of this technology to the clinic (11). Previously, we have generated hCMPs with thicknesses exceeding 2 mm *via* a novel layer-by-layer (LBL) method of hCMP assembly (12): hiPSC-derived cardiac cells were suspended in fibrinogen solution, and the solution was mixed with thrombin, deposited into a mold, and allowed to solidify before the procedure was repeated to produce two additional cell layers. In theory, an LBL manufacturing protocol could be used to produce hCMPs of any desired thickness by simply stacking the required number of cell layers (13); however, as hCMP thickness increases, cell viability tends to decline, because the diffusion of oxygen and nutrients from the media to interior of the hCMP is impaired. Thicker hCMPs may also be less well-vascularized by the endogenous circulatory system after transplantation and, consequently, methods for increasing vascularization, such as interleaving layers of endothelial cells between layers of cardiomyocytes (14), continue to be investigated.

For the experiments described here, we used a modified LBL fabrication procedure to generate thick LBL-hCMPs (~3.75 mm upon fabrication). Layers of hiPSC-CMs and hiPSC-derived endothelial cells (hiPSC-ECs) were generated separately and then sutured together, leaving gaps between the layers that allowed the medium to access the internal cell layer. LBL-hCMPs were also cultured on a rocking platform with electrical stimulation to promote hiPSC-CM maturation and connectivity, and the effectiveness of LBL-hCMP transplantation for promoting recovery from myocardial injury was evaluated in a mouse MI model.

## Materials and Methods

All procedures and protocols involving animals were approved by the Institutional Animal Care and Use Committee of the University of Alabama at Birmingham and performed in accordance with the National Research Council's Guide for the Care and Use of Laboratory Animals (NIH publication No 85–23).

### Differentiation of HiPSCs Into Cardiac Cells

Cardiac fibroblasts were isolated from the left atrium of an adult male patient and transfected with Sendai virus coding for OCT4, SOX2, KLF4, and C-MYC as described previously (15) to generate hiPSCs (LZ-hiPSC5), and the hiPSCs were maintained on Geltrex Membrane Matrix in mTeSR™ Plus basal and supplement medium. Reproducibility was ensured by utilizing another iPSC cell line (LW-hiPSC-V-2019). Cardiac fibroblasts were isolated from the ventricle of a male patient of 15 days old and transfected with Sendai virus coding for OCT4, SOX2, KLF4, and C-MYC. The pluripotency of LW-hiPSC-V-2019 was examined by teratoma formation (Data not shown). LW-hiPSC-V-2019 was used to generate the data in Supplementary Figure 2. LZ-hiPSC5 was used to generate all the other data.

The hiPSC-CM differentiation protocol was performed as described previously (16). Briefly, hiPSCs were cultured until 90–100% confluent, treated with CHIR99021 in RPMI basal medium plus B27 without insulin (B27–) on Day 0, recovered in RPMI basal medium plus B27– on Day 1, and then treated with IWR-1 in RPMI basal medium plus B27– on Day 3. The medium was replaced with RPMI basal medium plus B27– on Day 5; then, on Day 7 and every 3 days afterward, the medium was replaced with RPMI basal medium plus B27 with insulin (B27+). Beating cardiomyocytes usually appeared 7 days after differentiation was initiated, and the hiPSC-CMs were purified *via* metabolic selection (17) in glucose-free RPMI 1,640 medium (Gibco^TM^ Cat: 11879020) supplemented with 4 mM lactate and 2% B27+ for 6 days.

hiPSCs were differentiated into endothelial cells (hiPSC-ECs) as described previously (18). Briefly, hiPSCs were cultured until 90–100% confluent and treated with CHIR99021 (6–8 uM) supplemented in N2B27 medium for 3 days; then, the medium was replaced with StemPro-34 SFM medium containing vascular endothelial growth factor (VEGF, 200 ng/mL) and forskolin (2 uM), and the cells were cultured for 2 more days. hiPSC-ECs were purified *via* flow-cytometry selection for the expression of both CD31 and vascular endothelial cadherin (CD144).

### Surface-Marker Expression of HiPSC-Derived Cardiac Cells

The hiPSC-derived cardiac cells were characterized by immunofluorescence staining as described previously (5). Briefly, the cells were fixed with 4% paraformaldehyde (PFA) for 10 mins, permeabilized with 0.25% Triton X-100 for 15 mins, and then blocked with UltraV block (Thermo Scientific, USA) for 7 mins at room temperature. hiPSC-CMs were incubated with primary antibodies against cardiac troponin T (cTnT), cardiac troponin I (cTnI), and α sarcomeric actinin (αActinin), and hiPSC-ECs were incubated with primary antibodies against CD31, vascular endothelial cadherin (VE-cadherin), and von Willebrand factor (vWF); then, the cells were incubated with corresponding fluorescently conjugated secondary antibodies for 2 h, and nuclei were stained with 4,6-diamidino-2-phenyl-indole (DAPI). Fluorescent images were obtained with a confocal microscope.

### Flow Cytometry Analyses

The purity of hiPSC-CMs and -ECs was determined *via* flow cytometry analysis as described previously (11). Cells were trypsinized into single cells, fixed in 1% formaldehyde for 20 mins, permeabilized in 90% cold methanol for 15 mins, washed with 0.5% bovine serum albumin (BSA) in phosphate-buffered saline (PBS), and incubated with primary antibodies and isotype-control antibodies overnight at 4°C; then, the cells were incubated with fluorescent secondary antibodies for 30 mins, resuspended in 0.5% BSA in PBS, and evaluated with a FACS Aria instrument (BD Biosciences, USA).

### Fabrication of LBL- and Control-HCMPs

Individual cell layers containing either hiPSC-CMs or hiPSC-ECs were manufactured as described previously (11). Briefly, 2 million hiPSC-CMs (3 weeks after differentiation) or hiPSC-ECs (1 week after differentiation) were suspended in a fibrinogen solution; then, the cell-containing fibrinogen solution was mixed with a thrombin solution, and the mixture was quickly poured into a mold (internal dimensions: 1.5 × 1.5 cm; height: 1 cm) containing a nylon frame. The layers solidified within a few minutes and were cultured in Dulbecco minimum essential medium (DMEM) containing 10% fetal bovine serum (FBS), 1 × penicillin-streptomycin, and 2 mg/mL 6-aminocaproic acid (Thermo Fisher Scientific, Cat: AC103301000) for 24 h on a dynamic platform; then, individual cell layers were removed from the mold, and one hiPSC-EC layer was sandwiched between two hiPSC-CM layers, and the layers were sutured together with 8.0 surgical silk sutures by four stitches. The triple-layered LBL-hCMP was cultured in a six-well plate with electrical stimulation of 4 Hz, 6.5 V/cm, 5 ms pulse (C-Pace EP, IONOptix) on a rocking platform (45 rpm) for 2 weeks before *in vitro* characterization; synchronized beating was typically observed after 3 days of culture. Control-hCMPs consisted of 4 million hiPSC-CMs and 2 million hiPSC-ECs suspended in a single layer of fibrin and were fabricated *via* the same protocol used to produce the individual hiPSC-CM or hiPSC-EC layers; after manufacture, the Control-hCMPs were also cultured in DMEM containing 2% FBS, 1 × penicillin-streptomycin, and 2 mg/mL 6-aminocaproic acid on a rocking platform with electrical stimulation.

### HCMP Characterization

#### Immunofluorescence Staining

hCMPs were fixed in 4% PFA overnight at 4°C, embedded in optimal cutting temperature (OCT) compound, frozen, and cut into 10-μm sections; then, the sections were permeabilized with 0.25% Triton-X-100 for 15 mins, blocked with UltraV block (Thermo Scientific, USA) for 7 mins, and sequentially stained with primary antibodies (rabbit anti-cTnI, rabbit anti-Con43, mouse anti-cTnI, rabbit anti-N-Cadherin, rabbit anti-SERCA2, rabbit anti-RYR-2, rabbit anti-BIN1, mouse anti-Kir2.1 [Abcam, USA], mouse anti-α-sarcomeric actinin [Sigma-Aldrich, USA], rabbit anti-phosphorylated-MLKL [Cell signaling, USA], goat anti-CD31 [Santa Cruz Biotech, USA]) overnight at 4°C and with fluorescently conjugated secondary antibodies for 1 h at 37°C. Images were obtained with a fluorescent microscope.

#### Ca^2+^ Transients

Cardiomyocytes were dissociated from control hCMPs and LBL hCMPs with 0.2% collagenase type I (Fisher Scientific LS004194) in 20% FBS/DMEM (Gibco) at 37°C for 1 h, followed by gentle shaking with 0.25% trypsin/EDTA for 10 mins. Ca^2+^ transients were measured as described previously (19). Briefly, iPSC-CMs were incubated with Fura-2 AM (0.5 μM, Invitrogen, USA) for 10 mins in Tyrode's solution 2 days after dissociated iPSC-CMs were replated on cover glasses (25 × 25 mm) coated with Geltrex. The ratio of fluorescence emitted at 340 and 380 nm was recorded during 1 Hz field stimulation in Tyrode's solution with a Ca^2+^ recording system and analyzed with IonWizard (IonOptix, USA).

#### Quantitative Real-Time Polymerase Chain Reaction (qRT-PCR)

Total RNA was extracted with RNeasy mini kits (Qiagen, USA) as directed by the manufacturer's instructions; then, the RNA was quantified by Nanodrop and reverse transcribed with SuperScriptTM II Reverse Transcriptase (Thermo Scientific, USA). qRT-PCR was performed with Power Up SYBR Green PCR Mix (Thermo Fisher Scientific) and appropriate primers (Supplementary Table 1) on a QuantStudio 3 real-time PCR system (Eppendorf, USA). Measurements were determined *via* the 2^−ΔΔCt^ method and normalized to the abundance of glyceraldehyde phosphate dehydrogenase (GAPDH) RNA.

#### Transmission Electron Microscopy (TEM)

hCMPs were imaged *via* TEM in the the UAB High-Resolution Imaging Facility. Briefly, the hCMPs were fixed in 2.5% glutaraldehyde solution for 1 h at 4°C, embedded, and cut into ~70-nm sections with a diamond knife. Samples were mounted and photographed with a Tecnai Spirit T12 TEM.

#### Cardiomyocyte Alignment

Cardiomyocyte orientation and alignment was quantified as described previously (20). Briefly, hCMPs were immunostained with anti-cTnT and imaged with a confocal microscope. Cardiomyocyte orientation index was determined by analyzing anti-cTnT images in ImageJ using the Orientation J plug-in. Five independent samples were analyzed.

### Mouse MI Model and HCMP Implantation

MI was surgically induced in male and female NOD/SCID Gamma mice (stock #005557; The Jackson Laboratory) as described previously (8). Briefly, 12-week-old mice were anesthetized with inhaled isoflurane (1.5–2%), intubated, and ventilated with a small animal respirator; then, the heart was exposed *via* a left thoracotomy, and the left anterior descending coronary artery was permanently ligated with an 8–0 non-absorbable suture. Animals in the MI+LBL-hCMP group were treated with LBL-hCMPs, animals in the MI+Control-hCMP group were treated with Control-hCMPs, and animals in the MI group recovered without hCMP implantation. Animals in the sham group underwent all surgical procedures for MI induction except arterial ligation and recovered without hCMP implantation. A quarter of the LBL- or Control-hCMPs (due to the large size) were positioned over the infarcted region of the left ventricular (LV) anterior wall and sutured to the visceral layer of pericardium with two stitches; then, the chest was closed in layers, and mice were administered buprenorphine (0.1 mg/kg every 12 h for 3 consecutive days) and carprofen (5 mg/kg every 12 h for 1 day) for pain control during recovery.

### Echocardiographic Assessments of Cardiac Function

Echocardiographic measurements were performed as described previously (8). Briefly, mice were lightly anesthetized with 2% isofluorane until the heart rate stabilized at 400–500 beats/minute; then, B-mode and two-dimensional M-mode images of the heart were acquired from both the long-axis and short-axis views with a high-resolution micro-ultrasound system (Vevo 2100, VisualSonics, Inc.). At least eight consecutive images were analyzed, and LV internal diameters at end-diastole and end-systole (LVIDed and LVIDes, respectively) were determined with Vevo analysis software; then, LV ejection fraction (LVEF) and fractional shortening (LVFS) were calculated *via* the following equations:


$$\begin{array}{l} \text{LVEF}=\left({\text{LVIDed}}^{3}-{\text{LVIDes}}^{3}\right)/{\text{LVIDed}}^{3}\times 100\%\\ \text{LVFS}=\left(\text{LVIDed}-\text{LVIDes}\right)/\text{LVIDed}\times 100\% \end{array}$$

### Immunofluorescence Staining and Engraftment of Implanted HCMPs

Because the hiPSCs were of human origin (15), engrafted cells were identified by the expression of human nuclear antigen (HNA), engrafted hiPSC-CMs were identified by expression of the human variant of cardiac troponin T (hcTnT), and engrafted hiPSC-ECs were identified by the expression of human CD31 (hCD31). Hearts were fixed in 4% PFA overnight at 4°C, dehydrated with 30% sucrose, embedded in OCT compound, frozen, and cut into 10-μm sections; then, the sections were permeabilized with 0.25% Triton-X-100 for 35 mins, blocked with UltraV block (Thermo Scientific, USA) for 7 mins, and stained overnight at 4°C with Fluor-488–conjugated wheat germ agglutinin (WGA, Thermofisher Scientific, USA) and with primary antibodies (mouse anti-cTnT, rabbit anti-hcTnT, Abcam, USA; mouse anti-hCD31, BD Biosciences, USA; mouse anti-HNA, Emdmillipore, USA). Primary antibodies were labeled with fluorescently conjugated secondary antibodies for 1 h at 37°C, and the samples were photographed under a confocal microscope.

The engraftment rate was determined as described previously (8). Cells that expressed hcTnT and hCD31 were counted in every 20th section of the heart spanning the entire area covered by the engrafted patch, and the total was multiplied by 20 to determine the total number of hiPSC-derived cells per heart; then, the engraftment rate was calculated by dividing the total number of engrafted cells by a quarter of the number used during hCMP manufacture (1.5 × 10^6^) and expressed as a percentage.

CD31^+^ cell density of Control hCMP and LBL hCMP *in vitro* were assessed by counting the number of cells that expressed CD31. Assessments were conducted every 20th serial section from peripheral to middle, 5 fields *per section*. Arteriole density was evaluated by counting the number of structures that expressed αSMA, and hypertrophy was evaluated by calculating the cross-sectional surface area of cardiomyocytes as defined by the WGA stain (11); assessments were conducted in 5 sections per heart, 5 fields per section.

### Infarct Size and Wall Thickness Measurements

Infarct size and wall thickness were evaluated as described previously (8). Briefly, hearts were explanted, embedded in OCT compound, and cut into 10-μm sections from apex to base; then, every 13th serial section was fixed in Bouin's solution and stained with 0.04% sirius red to identify scar tissue and with 0.1% fast green to identify functional myocardial tissue. Sections were photographed and analyzed with Image J software. Infarct size was calculated as the ratio of the scar area to the total area of the LV, and infarct thickness was calculated as the ratio of the thicknesses of the fibrous region to the thickness of the septal wall; both ratios were presented as a percentage.

### Histochemistry

Frozen sections (10 μm) from animal hearts were stained in hematoxylin (Mayer's, Merck, 30 secs) and eosin Y (30 secs) solution, dehydrated, mounted in Permount, and imaged with a bright field microscope (Olympus IX83 epifluorescent microscope).

### Apoptosis

Apoptosis was evaluated with an *In-situ* Cell Death Detection Kit (Roche Applied Science, Germany) as described previously (11). Briefly, longitudinal sections from hCMPs and embedded hearts were fixed with 4% PFA, permeabilized with 0.25% Triton X-100, and stained with TUNEL solution for 1 h at 37°C as directed by the manufacturer's instructions; then, the samples were mounted in mounting medium with DAPI (Vector Laboratories) and viewed under a fluorescence microscope. Assessments were conducted every 13th serial longitudinal section per hCMP and 5 to 6 slides per animal, 5 fields per slide, and apoptosis was quantified as the ratio of the number of TUNEL-positive nuclei to the total number of nuclei.

### Statistical Analysis

Data were presented as mean ± SEM. Significance was determined *via* the Student's *t–*test for comparisons between two groups and *via* one-way analysis of variance for comparisons among three or more groups. A *p* value of <0.05 was considered statistically significant.

## Results

### LBL-HCMPs Were Thick With Greater Evidence of HiPSC-CM Maturation and Lower Rates of Apoptosis Than Were Observed in Control-HCMPs

**Figure 1:**
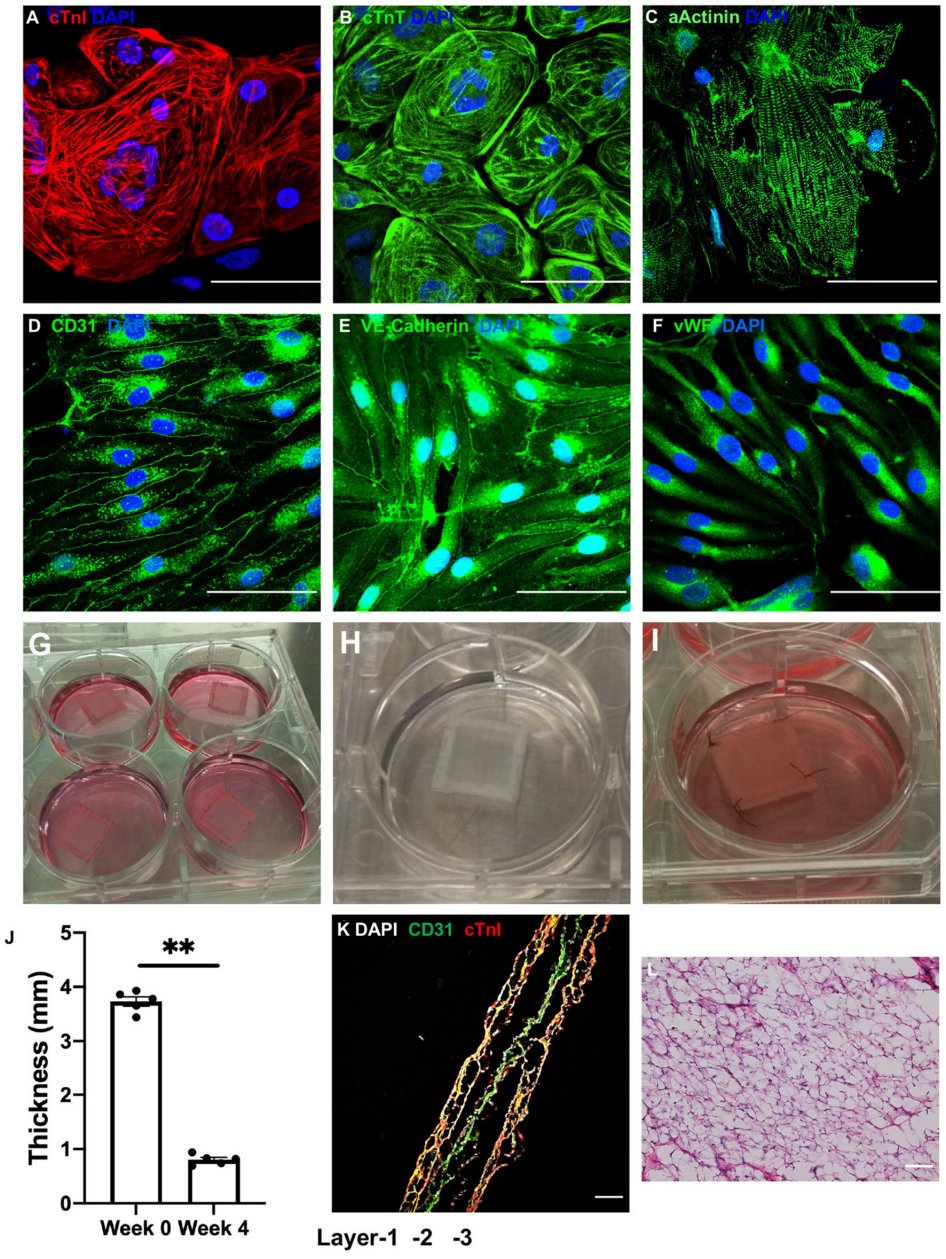
LBL-hCMPs were thick and composed of two layers of hiPSC-CMs surrounding a layer of hiPSC-ECs. **(A–C)** hiPSC-derived cardiomyocytes (hiPSC-CMs) were characterized *via* immunofluorescence analyses of **(A)** cardiac troponin I (cTnI), **(B)** cardiac troponin T (cTnT), and **(C)** α sarcomeric actinin (αActinin) expression; and **(D–F)** hiPSC-derived endothelial cells (hiPSC-ECs) were characterized *via* immunofluorescent analyses of **(D)** CD31, **(E)** vascular endothelial cadherin (VE-cadherin), and **(F)** von Willebrand factor (vWF) expression. Nuclei were counterstained with 4',6-diamidino-2-phenylindole (DAPI); bar = 100 μm. **(G–I)** LBL hCMPs were composed of two layers of hiPSC-CMs and one layer of hiPSC-ECs. The individual **(G)** hiPSC-EC and **(H)** hiPSC-CM layers were fabricated by suspending 2 million cells of the corresponding type in a fibrinogen solution, mixing the cell-containing fibrinogen solution with a thrombin solution, quickly pouring the mixture into a mold (internal dimensions: 1.5 cm × 1.5 cm; height: 1 cm) containing a nylon frame, and culturing the cell layer for one day on a dynamic platform; then, individual cell layers were removed from the mold for fabricating LBL-hCMPs. **(I)** A layer of hiPSC-ECs was sandwiched by two layers of hiPSC-CMs; then, the layers were sutured together with four stitches and cultured in a 6-well plate on a rocking platform with electrical stimulation. **(J)** LBL-hCMP thickness was measured with a caliper immediately after fabrication (Week 0) and at Week 4. (*n* = 5, ^**^P < 0.01). **(K)** The LBL-hCMPs were cut into longitudinal sections, and the internal structure was evaluated by staining for CD31 and cTnI; nuclei were counter-stained with DAPI (bar = 200 μm). **(L)** The patch was cut in cross-section, and the internal structure was evaluated *via* hematoxylin/eosin staining; bar = 100 μm.

hiPSCs were reprogrammed from human cardiac fibroblasts and differentiated into hiPSC-CMs and -ECs. hiPSC-CMs (Figures 1A–C) expressed the CM-specific markers cardiac troponin I (cTnI), cardiac troponin T (cTnT), and α sarcomeric actinin (αActinin); and hiPSC-ECs (Figures 1D–F) expressed the EC-specific markers CD31, vascular endothelial cadherin (VE-cadherin), and von Willebrand factor (VWF). Flow cytometry analysis confirmed that both hiPSC-derived cell populations were >95% pure: 97.0% of the hiPSC-CMs expressed cTnT (Supplementary Figure 1A), and 99.9% of the hiPSC-ECs expressed VE-cadherin (Supplementary Figure 1B).

The LBL-hCMPs were composed of a single layer of hiPSC-ECs and two layers of hiPSC-CMs. Individual cell layers were produced by mixing a fibrinogen solution containing 2 million hiPSC-CMs or hiPSC-ECs with thrombin, and then quickly pouring the mixture into a mold (internal dimensions: 1.5 cm × 1.5 cm; height: 1 cm) containing a nylon frame. The mixture solidified within a few minutes and was cultured for 24 h on a dynamic platform; then, individual cell layers were removed from the mold, and the hiPSC-EC layer (Figure 1G) was sandwiched between the hiPSC-CM layers (Figure 1H), the three layers were sutured together (Figure 1I), and the LBL-hCMP was cultured on a rocking platform (45 rpm) with electrical stimulation of 4 Hz, 6.5 V/cm, 5 ms pulse (C-Pace EP, IONOptix) for 2 weeks. Synchronous beating (Supplementary Video) was typically observed 3 days later, and the thickness of the LBL-hCMP declined from 3.73 ± 0.09 mm immediately after layering to 0.80 ± 0.04 mm after 4 weeks of culture (Figure 1J). Control-hCMPs (1.5 × 1.5 cm) were fabricated by combining 4 million hiPSC-CMs and 2 million hiPSC-ECs into a single cell layer and were cultured on a rocking platform with electrical stimulation. Control-hCMPs were 3.65 ± 0.08 mm thick immediately after manufacture and 0.76 ± 0.04 mm after the 4-week culture period (Supplementary Figure 2C).

Analysis of LBL-hCMP cross-sections that had been immunofluorescently stained for cTnI and CD31 (Figure 1K) indicated that some hiPSC-ECs had migrated into the hiPSC-CM layer. Notably, LBL-hCMP sections stained with hematoxylin and eosin (Figure 1L) suggests that all cells within the LBL-hCMP were accessed by the culture medium.

**Figure 2:**
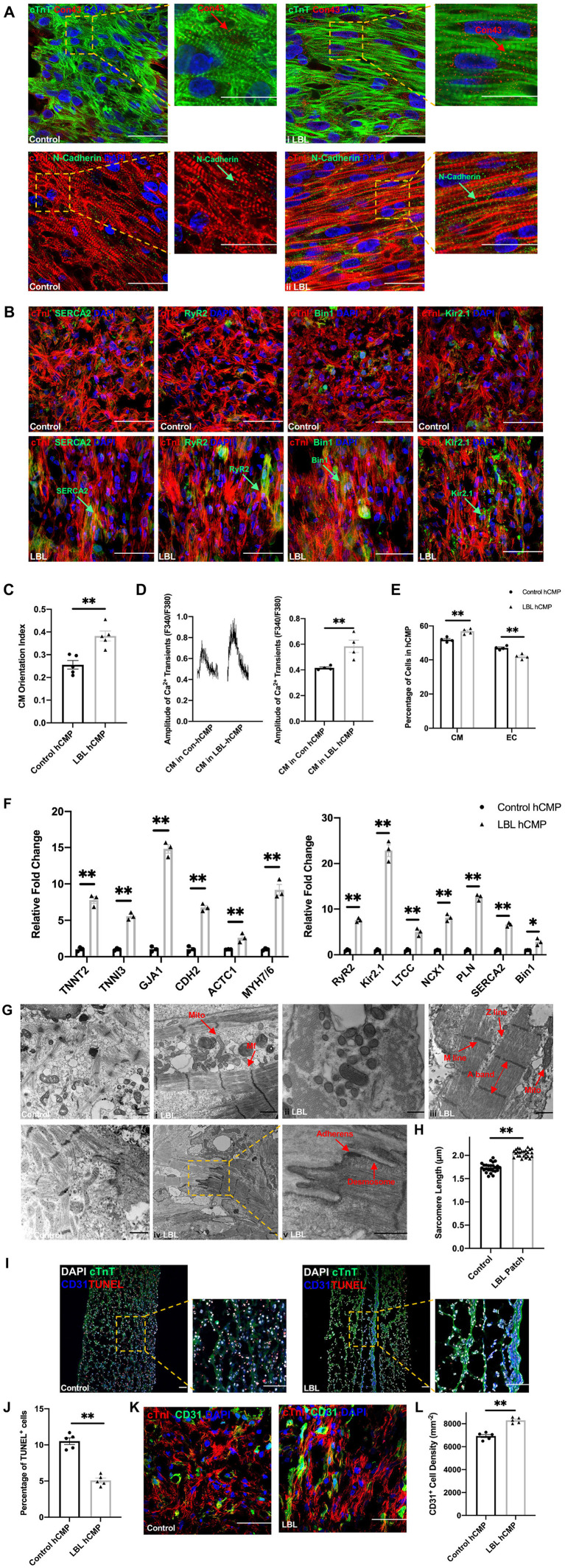
hiPSC-CMs were more mature, and apoptotic cells were less common, in LBL-hCMPs than in Control-hCMPs. **(A,B)** The morphology of hiPSC-CMs in Control- and LBL-hCMPs was evaluated in **(A)** sections stained for the expression of cardiac troponin T (cTnT), cardiac connexin 43 (Con43), cardiac troponin I (cTnI), and N-Cadherin; and in **(B)** sections stained for the expression of cTnI, SERCA2, RyR2, BIN1, and Kir2.1. **(C)** Cardiomyocyte orientation index was assessed to compare cardiomyocyte alignment between Control-hCMP and LBL-hCMP (*n* = 5). **(D)** Dissociated hiPSC-CMs were incubated with Fura-2, a Ca^2+^ indicator; then transients were recorded with continuous 1 Hz electric stimulation, and transient amplitudes were calculated. **(E)** The percentage of iPSC-CMs and -ECs were determined by quantifying cells that stained positively for each marker (*n* = 4 hCMPs). **(F)** The expression of genes coding for cardiomyocyte structural and contractile proteins (left) and calcium-handling proteins (right) was evaluated in Control- and LBL-hCMPs *via* quantitative RT-PCR assessments of mRNA abundance; results were normalized to measurements in Control-hCMPs. **(G)** hCMP ultrastructure was analyzed by transmission electron microscopy (TEM); myofibrils (Mf), mitochondria (Mito), A-bands, M-lines, Z-lines, fascia adherens junctions (Adherens) and desmosomes are identified with arrows (bars = 1 μm). **(H)** Sarcomere lengths were measured in TEM images (*n* = 25). **(I)** Sections from Control- and LBL-hCMPs were stained for the expression of cTnT and CD31, apoptotic cells were identified *via* terminal deoxynucleotidyl transferase dUTP nick end labeling (TUNEL), and nuclei were counterstained with DAPI (bar = 100 μm); then, **(J)** apoptosis was quantified as the percentage of TUNEL^+^ cells (*n* = 5). **(K)** Sections from Control-hCMPs and LBL-hCMPs were stained for the expression of cTnI and CD31, nuclei were counter-stained with DAPI, and **(L)** CD31^+^ cell density was calculated by quantifying the number of cells that expressed CD31. (*n* = 5). Assessments were performed in hCMPs that had been cultured for 14 days in panels A-K. *P < 0.05, **P < 0.01.

Morphological assessments of hCMPs that had been immunofluorescently stained for cTnT or cTnI, and for the gap-junction protein Connexin 43, the adhesion protein N-Cadherin (Figure 2A), and the calcium handling proteins SERCA2, RYR2, Bin1, and Kir2.1 (Figure 2B), together with quantification of CM orientation index (Figure 2C) and intracellular calcium transients (Figure 2D), suggested that hiPSC-CMs were better aligned, and the calcium-handling machinery was more robust, in LBL-hCMPs than in control hCMPs. The proportion of iPSC-CMs and -ECs were calculated (Figure 2E), and mRNA transcripts for proteins that participate in CM contractile activity (TNNT2, TNNT3, GJA1, CDH2, ACTC1, MYH7/6) and in generating calcium transients (RYR2, KIR2.1, LTCC, NCX1, PLN, SERCA2, BIN1) were also significantly more abundant in LBL- than in Control-hCMPs (Figure 2F), and when imaged *via* transmission electron microscopy, the sarcomeres of LBL-hCMPs appeared well-ordered with visible A-bands, M-lines, and Z-lines, as well as primitive intercalated disc-like structures that contained fascia adherens junctions and desmosomes (Figure 2G). Sarcomeres were also significantly longer in LBL-hCMPs (2.053 ± 0.015 μm) than in Control-hCMPs (1.738 ± 0.020 μm) (Figure 2H), and LBL-hCMPs contained a smaller proportion of apoptotic cells (Figures 2I,J). CD31^+^ cell density in the LBL-hCMPs was significantly higher compared to Control-hCMP after 2 weeks of culture (Figures 2K,L), which suggests that the LBL-hCMPs were primed for *in-vivo* neovascularization.

### The Layered Structure of LBL-HCMPs Was Maintained After Implantation Into Infarcted Mouse Hearts

**Figure 3:**
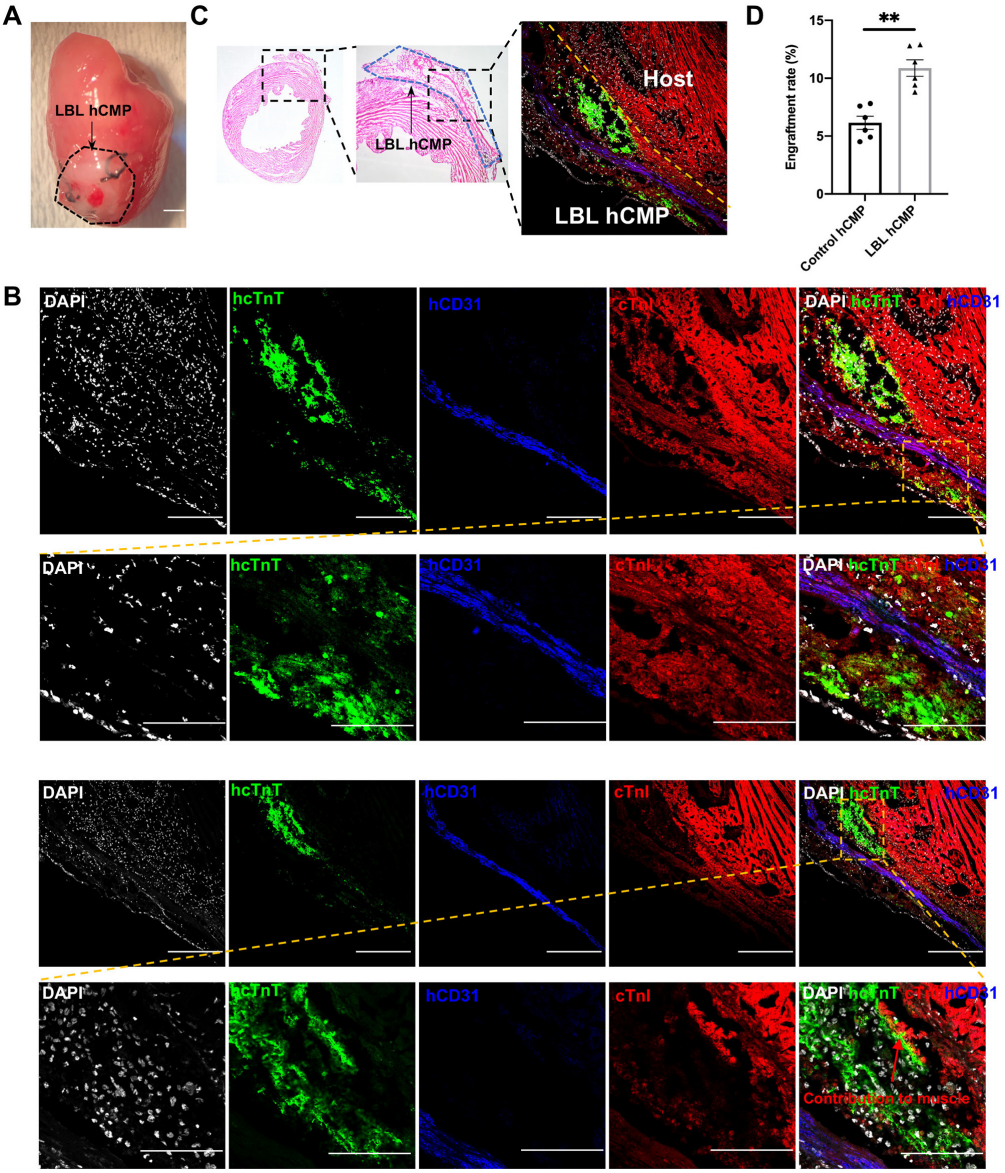
Individual layers of the LBL-hCMPs remained intact after implantation into infarcted mouse hearts. MI was surgically induced in mice, and **(A)** LBL-hCMPs were sutured over the site of infarction; bar = 1 mm. **(B,C)** 28 days later, sections from the LBL-hCMP-treated hearts were stained for the human isoform of cTnT (hcTnT) to identify hiPSC-CMs, for human CD31 (hCD31) to identify hiPSC-ECs; bar = 100 μm. **(C)** The representative Hematoxylin and eosin (HE) staining image and immunostaining image demonstrated the layered structure of the implanted hCMP remained visible to the left of the dashed yellow line. **(D)** The number of cells that expressed hcTnT and hCD31 was divided by a quarter of the total number of cells used during hCMP manufacture (1.5 × 10^6^) and expressed as a percentage; assessments were conducted in LBL hCMP and Control hCMP group 4 weeks after transplantation. *P < 0.05, **P < 0.01 (*n* = 6 hearts).

The cardioprotective efficacy of LBL- and Control-hCMPs was compared in a mouse model of MI. MI was surgically induced by permanently ligating the left anterior descending coronary artery, and the animals were randomly distributed into three groups. Animals in the MI+LBL-hCMP and MI+Control-hCMP groups were treated with LBL- or Control-hCMPs, respectively, and animals in the MI group recovered without receiving either hCMP construct; the hCMPs were positioned over the site of infarction and held in place with two sutures (Figure 3A). A fourth group of animals (the Sham group) underwent all surgical procedures for MI induction except the ligation step and recovered without either experimental treatment (Supplementary Table 2).

Because the transplanted cells were of human origin, engraftment was evaluated *via* immunofluorescence staining for human- and lineage-specific markers: hiPSC-CMs were identified by expression of the human variant of cardiac troponin T (hcTnT), and hiPSC-ECs were identified by the expression of human-specific CD31 (hCD31) (Figure 3B). Observations in the hearts of LBL-hCMP animals indicated that the layer-by-layer structure of the LBL-hCMP remained intact 4 weeks after implantation (Figure 3C), and that the engraftment rate for the combined hiPSC-CM and -EC populations in LBL hCMP group (10.88 ± 0.71%) was significant higher compared to that in Control hCMP group (6.15 ± 0.57%) at Week 4 (Figure 3D).

### LBL-HCMPs Were More Potent Than Control-HCMPs for Promoting Myocardial Recovery and Vascularization

**Figure 4:**
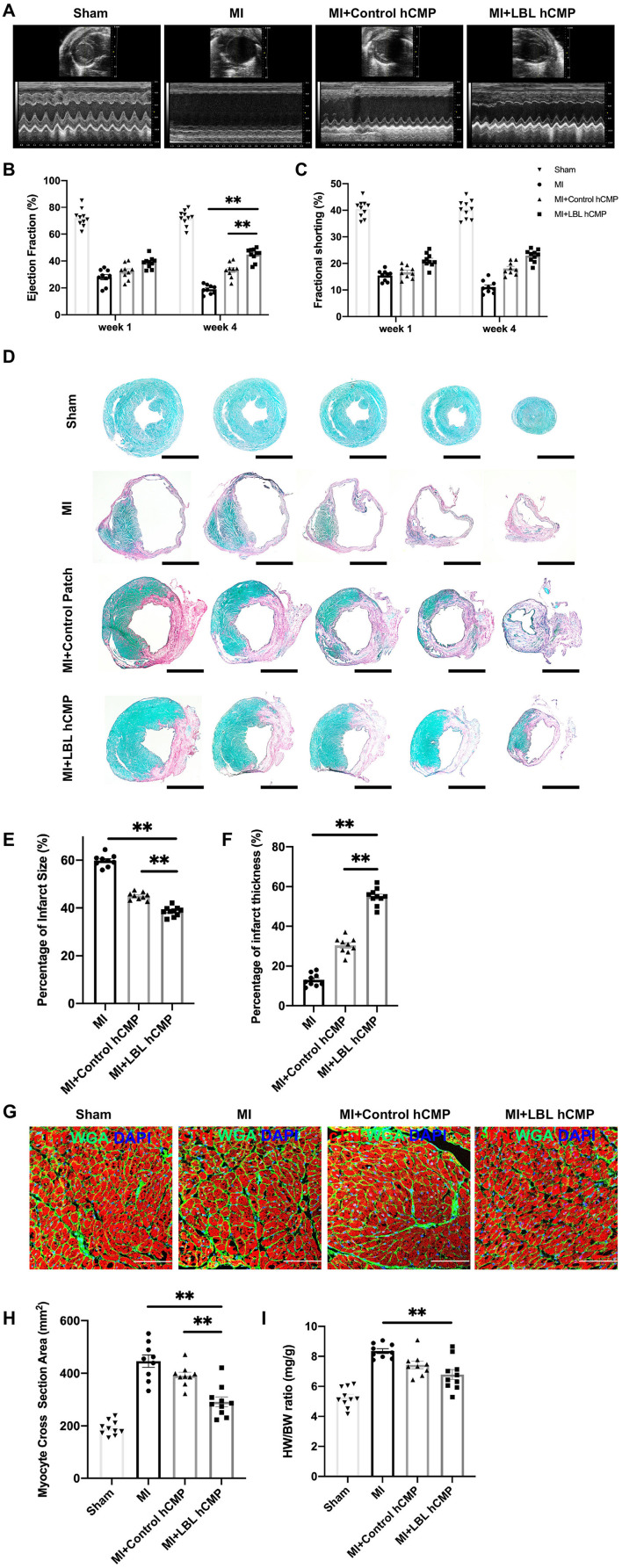
Measures of cardiac function, infarct size, and hypertrophy were better in mice treated with LBL-hCMPs after MI than in the Control-hCMP treatment group. MI was induced in mice by ligating the left-anterior descending coronary artery, and the animals were treated with LBL-hCMPs (the MI+LBL-hCMP group), with Control-hCMPs (the MI+Control-hCMP group), or with neither of the hCMP constructs (the MI group). Animals in the Sham group underwent all surgical procedures for MI induction except the ligation step and recovered without either experimental treatment. **(A)** Echocardiographic assessments of **(B)** left ventricular ejection fraction and **(C)** fractional shortening were performed at Week 1 and Week 4 after MI. **(D–H)** Sections from hearts explanted at Week 4 were **(D)** stained with picro-sirius red; scar tissue appears red, and functional myocardium appears green. Scale bar: 1 mm. **(E)** Infarct size was calculated as the ratio of the area of the scar to the total surface area of the left ventricle and presented as a percentage. **(F)** Infarct wall thickness was calculated as the ratio of the thickness of the scar to the thickness of the septal wall and presented as a percentage. **(G,H)** Heart sections were cut perpendicular to the long axis of the cardiac muscle fibers and then **(G)** stained for cTnI expression (red) to visualize the muscle fibers and with wheat germ agglutinin (WGA) to identify the borders of the cardiomyocytes; nuclei were counter-stained with DAPI. **(H)** Cardiomyocyte hypertrophy was evaluated by calculating the cardiomyocyte cross-sectional surface area. **(I)** Cardiac hypertrophy was evaluated at Week 4 by calculating the ratio of the heart-weight to bodyweight (HW/BW) for each animal. *n* = 9–10 animals per group; *P < 0.05, **P < 0.01. Bar = 100 μm.

**Figure 5:**
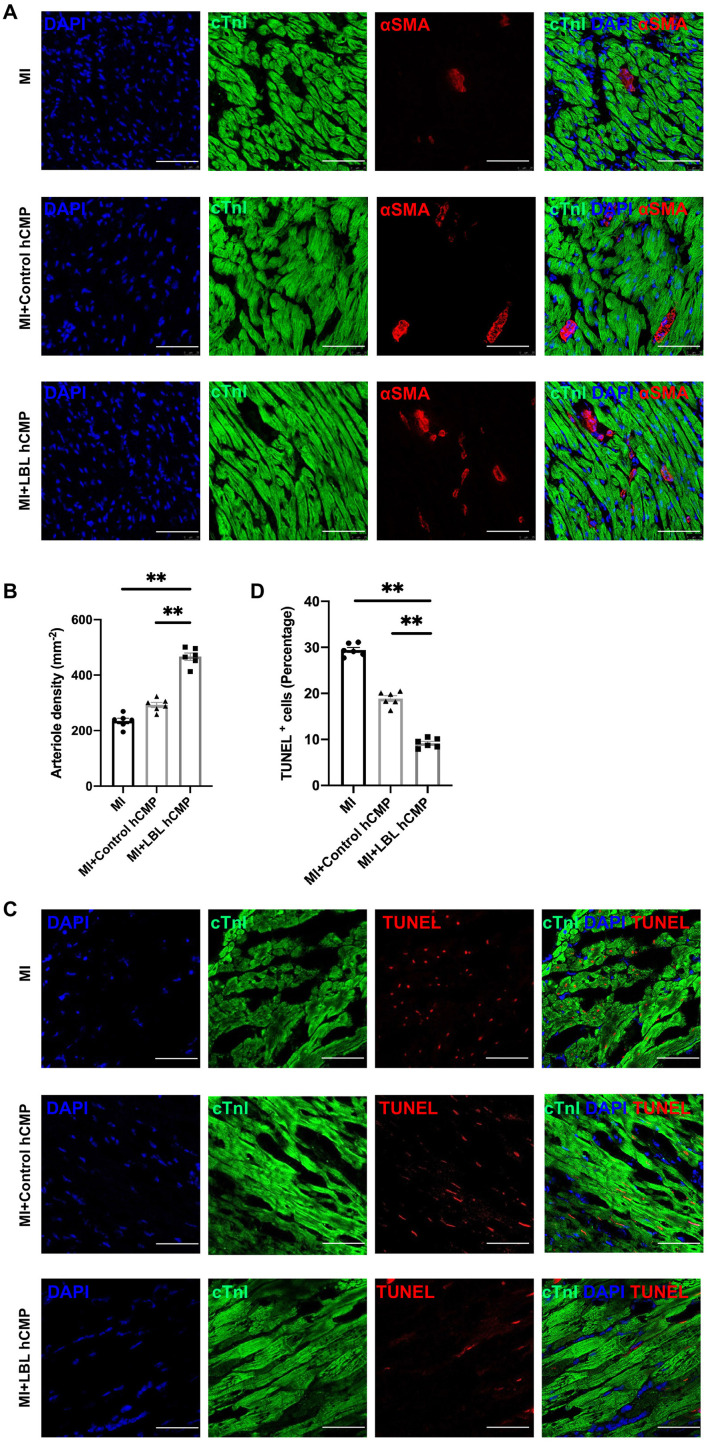
Vascular density was greater, and apoptotic cells were less common, in the hearts of MI+LBL-hCMP animals than in the hearts of MI+Control-hCMP animals. Sections from the border zone of the infarct were collected from the hearts of animals in the MI, MI+Control-hCMP, and MI+LBL-hCMP groups 4 weeks after MI. **(A)** Sections from border zone of the infarct were stained for the expression of αSMA and cTnI (bar = 200 μm), and nuclei were counterstained with DAPI; then, **(B)** arteriole density was determined by quantifying the number of vessel-like structures that expressed αSMA. **(C)** Sections from border zone of the infarct were stained for the expression of cTnI, apoptotic cells were identified *via* TUNEL staining, and nuclei were counterstained with DAPI (bar = 100 μm); then, **(D)** Cardiomyocyte apoptosis was quantified as the percentage of cTnT-positive cells that were also positive for TUNEL. *n* = 6 in each group, *P < 0.05, ^**^P < 0.01.

Cardiac function was evaluated one and 4 weeks after MI induction or sham surgery *via* echocardiographic assessments (Figure 4A) of left-ventricular ejection fraction (LVEF, Figure 4B) and fractional shortening (LVFS, Figure 4C): both parameters were significantly greater in MI+LBL-hCMP animals than in the MI+Control-hCMP or MI groups at Week 4. Furthermore, measurements of infarct size and wall thickness (Figures 4D–F), cardiomyocyte cross-sectional surface area (Figures 4G,H), heart-weight-to-bodyweight ratio (Figure 4I), the density of αSMA-expressing vascular structures (Figures 5A,B), and the proportion of TUNEL-positive cells (Figures 5C,D) were all significantly better at Week 4 after treatment with LBL-hCMPs than in the MI+Control-hCMP or MI groups. Thus, LBL-hCMP implantation was associated with significantly greater improvements in myocardial function, hypertrophy, vascularity, and apoptosis.

## Discussion

The goal of cardiac tissue engineering is to develop hCMPs that fully replicate the structural and functional properties of the native myocardium (21). However, even technologically sophisticated production methods, such as multiphoton-excited three-dimensional printing technology (8), tend to produce hCMPs that are too thin and functionally immature for therapeutic applications (22). hCMPs with clinically relevant surface areas (4 cm × 2 cm) significantly improved cardiac function and reduced infarct size when evaluated in a swine model of myocardial injury (11), but the constructs were just 1.25 mm thick immediately after fabrication. Thus, the LBL assembly method presented in this report may facilitate the clinical translation of engineered myocardial tissues by enabling the production of substantially thicker (3.75 mm) constructs with no decline in cell viability. hiPSC-CMs were also more mature in the LBL-hCMPs than in Control-hCMPs, and measurements of engraftment, cardiac function, infarct size, and hypertrophy were significantly better in mice treated with LBL-hCMPs after MI than in Control-hCMP-treated animals.

hCMPs with thicknesses exceeding 2 mm have also been produced by depositing 2–3 layers of cell-containing fibrin solution into a single mold, and allowing each layer to polymerize before adding the subsequent layer (12). However, the thickness of an hCMP is limited primarily by the diffusion of oxygen and nutrients from the culture medium. Thus, since our LBL-hCMPs were fabricated by suturing the individual cell layers together and then cultured on a rocking platform, the gaps between layers during each contraction ensured that each layer was maximally perfused by the medium. Consequently, the apoptosis rate in LBL hCMP was significantly lower compared to Control hCMP after 2-week culture. Notably, the layered structure of the LBL-hCMP remained intact after implantation, and the engraftment rate was significantly greater for LBL-hCMPs than for Control-hCMPs, which may suggest that EC-containing hCMPs are more efficiently vascularized by the endogenous circulatory system when the ECs are distributed in a single layer, rather than homogenously dispersed throughout the hCMP. Vascularized iPSC-CM 3D tissues have been successfully fabricated with cell accumulation technique and proved to promote development of capillary network when evaluated in a rat model of myocardial infarction (23). Filtration-Layer-by-Layer (LBL) technique has been utilized to generate vascularized 3D-iPSC-CM tissues, demonstrating significant different toxicity responses compared with 2D-iPSC-CM cells (24). Vascularization has also been enhanced by interleaving contiguous sheets of vascular cells with monolayers of cardiomyocytes (14), but cells within the individual sheets were more extensively interconnected with each other than with cells in adjacent sheets, likely because the sheets were generated by culturing a population of seeded cells for several days before hCMP assembly. The disparity between inter- and intra-layer connectivity was likely lower in our LBL-hCMPs, because the cells were suspended in a fibrin matrix, and the layers were cultured for just 24 h before assembly. Intra-layer connectivity can also be improved by constructing hCMPs with graphene-oxide–based thin films (25) or a tissue-velcro platform (26).

hiPSC-CMs are phenotypically more similar to fetal than adult cardiomyocytes and, consequently, engineered cardiac tissues tend to be less mature than the myocardium of adult animals (3). A variety of techniques have been developed to promote the maturation of hCMPs (27), including electrical pacing, mechanical stimulation (28), and culturing the hCMPs under dynamic conditions (11). For the studies presented here, the LBL-hCMPs were cultured with electrical stimulation on a rocking platform, which promoted cellular alignment and the development of mature sarcomeric structures, as well as the expression of genes that participate in calcium handling and contractile activity. The gaps between different layers guaranteed the survival of separate cell populations while control hCMPs demonstrated necrotic core due to nutrient and oxygen diffusion limitation after culturing for 2 weeks in these experimental settings. Maturation could also have been enhanced by paracrine factors released from the hiPSC-ECs, while electrical stimulation may have improved coupling between the two hiPSC-CM layers, which could reduce electromechanical heterogeneity and the subsequent risk of arrhythmia after implantation. Arrhythmias were not reported when sheets of autologous myoblasts were evaluated in a patient with dilated cardiomyopathy (29) but have been observed in non-human primates after the administration of cardiomyocytes differentiated from embryonic stem cells (30, 31) and, consequently, continue to be perhaps the most prominent safety concern associated with cardiomyocyte transplantation (32). Measures of electrical instability and arrythmogenesis were not evaluated in the studies reported here, but there were no incidents of sudden death in any treated animals.

In conclusion, our modified LBL protocol enabled us to generate thicker hCMPs with no loss of cell viability. hiPSC-CMs were also more mature in LBL-hCMPs than in Control-hCMPs, and measures of cardiac function, infarct size, hypertrophy, vascularity, and apoptosis were significantly better in mice treated with LBL-hCMPs after surgically induced MI than in the Control-hCMP treatment group. Collectively, these observations indicate that future investigations in large-animal models of myocardial injury are warranted to evaluate the potential benefit of LBL-hCMP implantation for promoting myocardial repair, and to more thoroughly characterize potential safety concerns, such as the risk of arrhythmogenic complications.

## Data Availability Statement

The original contributions presented in the study are included in the article/Supplementary Materials, further inquiries can be directed to the corresponding author/s.

## Ethics Statement

The animal study was reviewed and approved by Institutional Animal Care and Use Committee of the University of Alabama at Birmingham.

## Author Contributions

LW and JZ: designed the project, wrote the manuscript, and revised the manuscript. LW: conducted all the experiments. JZ; supervised the whole project. Both the authors approved the submission and publication of the manuscript.

## Funding

This work was supported in part by the following funding sources: NIH RO1s, HL114120, HL 131017, HL 149137, NIH UO1 HL134764, and AHA 20PRE35210006.

## Conflict of Interest

The authors declare that the research was conducted in the absence of any commercial or financial relationships that could be construed as a potential conflict of interest.

## Publisher's Note

All claims expressed in this article are solely those of the authors and do not necessarily represent those of their affiliated organizations, or those of the publisher, the editors and the reviewers. Any product that may be evaluated in this article, or claim that may be made by its manufacturer, is not guaranteed or endorsed by the publisher.

## Supplementary Material

Supplementary Figure 1 hiPSC-CM and -EC populations were at least 97% pure. The purity of the **(A)** hiPSC-CM and **(B)** hiPSC-EC populations was evaluated *via* flow cytometry analysis of cTnT and VE-cadherin expression, respectively (blue: cTnT or VE-cadherin; red: control).

Supplementary Figure 2 Fabrication and *in vitro* characterization of LBL-hCMP with another iPSC cell line (LW-hiPSC-V-2019) (Data not shown). **(A)** The LBL-hCMPs fabricated with Ven-FB-iPSC were cut into longitudinal sections, and the internal structure was evaluated by staining for CD31 and cTnI; nuclei were counter-stained with DAPI (bar = 200 μm). **(B)** The morphology of hiPSC-CMs in LBL hCMPs was evaluated in sections stained for the expression of cardiac troponin I (cTnI), and N-Cadherin. **(C)** Control-hCMP thickness was measured with a caliper immediately after fabrication (Week 0) and at Week 4. (*n* = 5, ^*^^*^*P* < 0.01). **(D)** Cardiomyocyte orientation index was assessed to compare cardiomyocyte alignment between Control-hCMP and LBL-hCMP (*n* = 4). **(E)** Sections from Control- and LBL-hCMPs were stained for the expression of cTnT and CD31, apoptotic cells were identified *via* terminal deoxynucleotidyl transferase dUTP nick end labeling (TUNEL), and nuclei were counterstained with DAPI (bar = 100 μm); then, **(F)** apoptosis was quantified as the percentage of TUNEL^+^ cells (*n* = 3).

Supplementary Figure 3 **(A)** The representative Hematoxylin and eosin (HE) staining image demonstrated sacrificed mouse heart after Control-hCMP transplantation. Delta left ventricular ejection fraction **(B)** and delta fractional shortening **(C)** pre-transplant to 4 weeks were assessed. *n* = 9–10 animals per group; ^*^*P* < 0.05, ^*^^*^*P* < 0.01. Bar = 100 μm.

Supplementary Table 1 Primers for quantitative RT-PCR.

Supplementary Table 2 Number of animals in each group.

Supplementary Video Synchronized beating of hiPSC-CMs in LBL-hCMPs on Day 7 after layering.
