# Supplementary figures and images for: Layer-By-Layer Fabrication of Thicker and Larger Human Cardiac Muscle Patches for Cardiac Repair in Mice
Source: Front Cardiovasc Med. 2022 Jan 6;8:800667. doi: 10.3389/fcvm.2021.800667 (PMC8770979; doi:10.3389/fcvm.2021.800667)

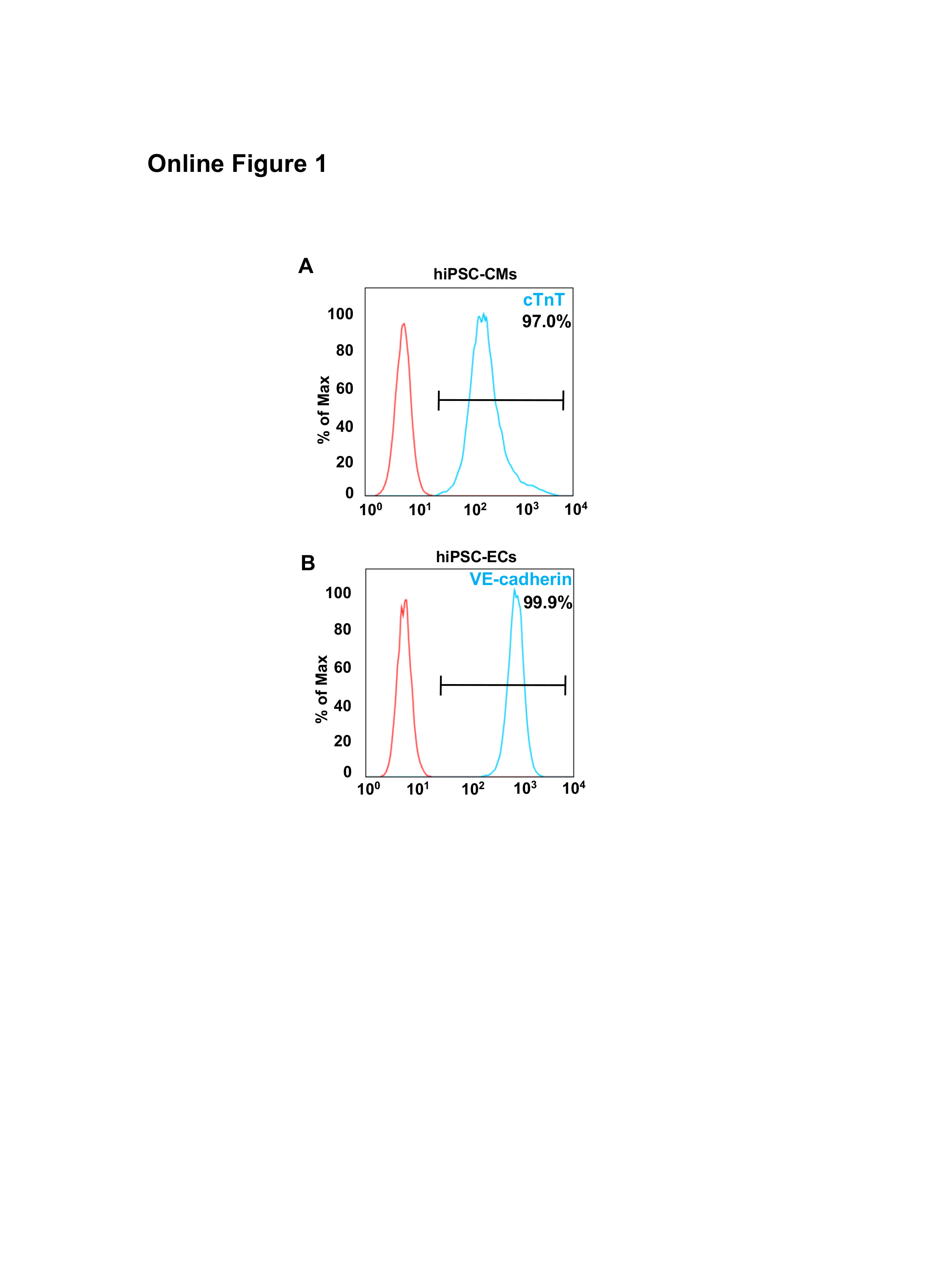

Supplement: Supplementary Figure 1 — hiPSC-CM and -EC populations were at least 97% pure. The purity of the (A) hiPSC-CM and (B) hiPSC-EC populations was evaluated via flow cytometry analysis of cTnT and VE-cadherin expression, respectively (blue: cTnT or VE-cadherin; red: control). [file Image_1.JPEG]

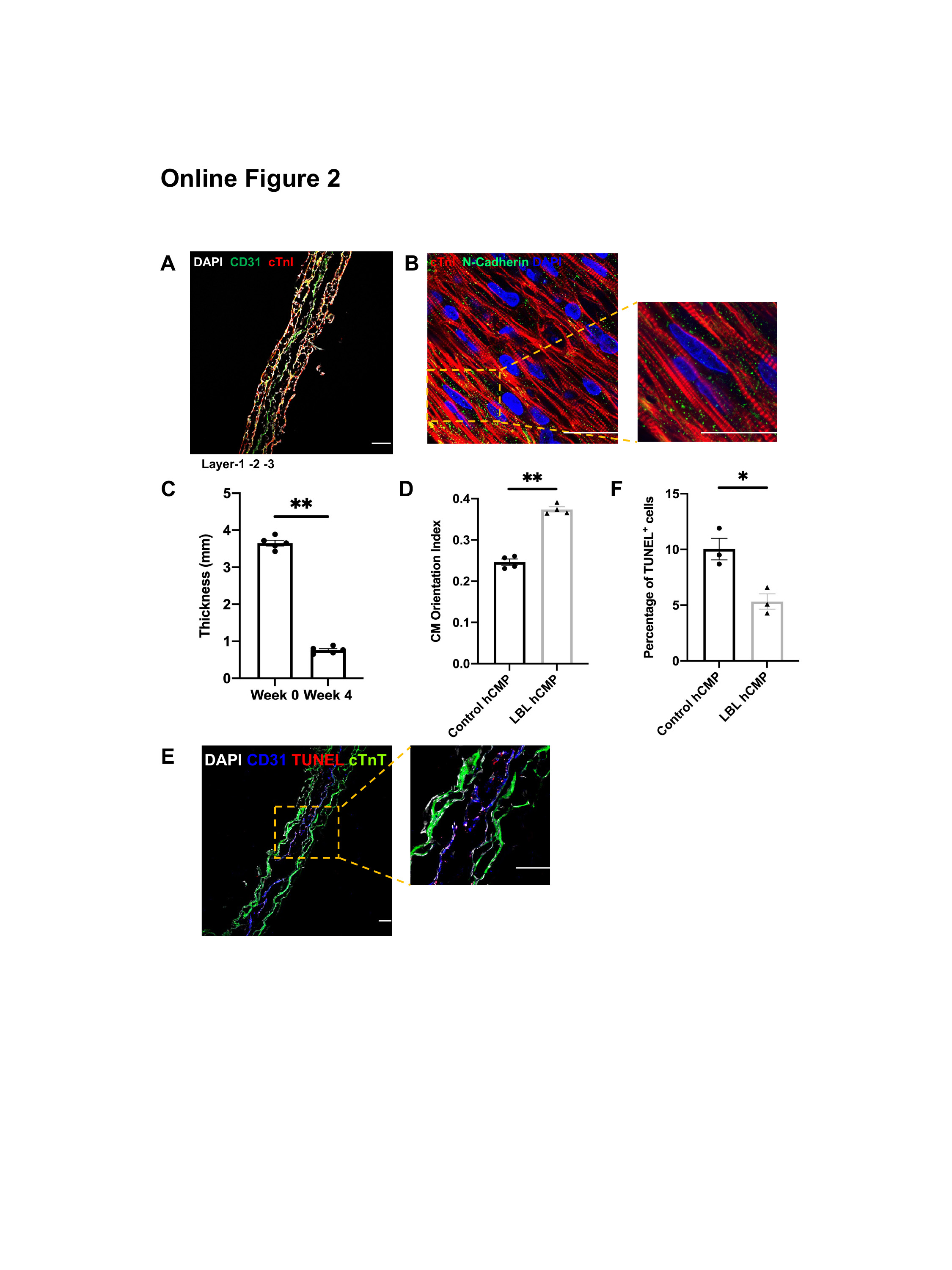

Supplement: Supplementary Figure 2 — Fabrication and in vitro characterization of LBL-hCMP with another iPSC cell line (LW-hiPSC-V-2019) (Data not shown). (A) The LBL-hCMPs fabricated with Ven-FB-iPSC were cut into longitudinal sections, and the internal structure was evaluated by staining for CD31 and cTnI; nuclei were counter-stained with DAPI (bar = 200 μm). (B) The morphology of hiPSC-CMs in LBL hCMPs was evaluated in sections stained for the expression of cardiac troponin I (cTnI), and N-Cadherin. (C) Control-hCMP thickness was measured with a caliper immediately after fabrication (Week 0) and at Week 4. (n = 5, **P < 0.01). (D) Cardiomyocyte orientation index was assessed to compare cardiomyocyte alignment between Control-hCMP and LBL-hCMP (n = 4). (E) Sections from Control- and LBL-hCMPs were stained for the expression of cTnT and CD31, apoptotic cells were identified via terminal deoxynucleotidyl transferase dUTP nick end labeling (TUNEL), and nuclei were counterstained with DAPI (bar = 100 μm); then, (F) apoptosis was quantified as the percentage of TUNEL+ cells (n = 3). [file Image_2.JPEG]

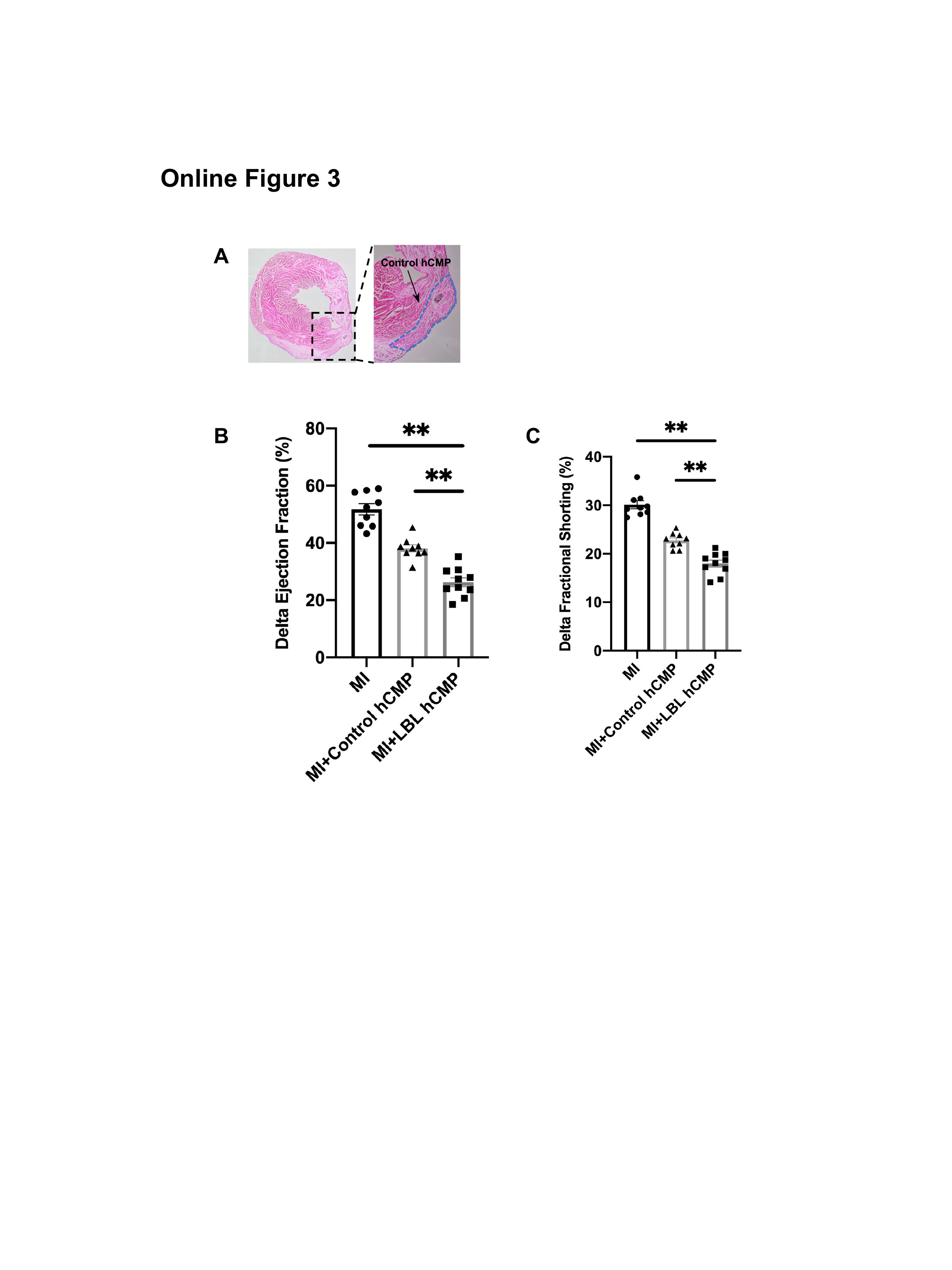

Supplement: Supplementary Figure 3 — (A) The representative Hematoxylin and eosin (HE) staining image demonstrated sacrificed mouse heart after Control-hCMP transplantation. Delta left ventricular ejection fraction (B) and delta fractional shortening (C) pre-transplant to 4 weeks were assessed. n = 9–10 animals per group; *P < 0.05, **P < 0.01. Bar = 100 μm. [file Image_3.JPEG]
