# Supplementary material for: Layer-By-Layer Fabrication of Thicker and Larger Human Cardiac Muscle Patches for Cardiac Repair in Mice
Source: Front Cardiovasc Med. 2022 Jan 6;8:800667. doi: 10.3389/fcvm.2021.800667 (PMC8770979; doi:10.3389/fcvm.2021.800667)
Supplement: Supplementary Table 1 — Primers for quantitative RT-PCR. [file Data_Sheet_1.docx]

**Supplemental Table 1.** Primers for quantitative RT-PCR

| **Standard gene name** | **Gene product** | **Forward 5’-3’** | **Reverse 5’-3’** |
| --- | --- | --- | --- |
| TNNT2 | cTnT | TTCACCAAAGATCTGCTCCTCGCT | TTATTACTGGTGTGGAGTGGGTGTGG |
| TNNI3 | cTnI | CCTCACTGACCCTCCAAACG | GAGGTTCCCTAGCCGCATC |
| GJA1 | CX43 | GGTCTGAGTGCCTGAACTTGCCT | AGCCACACCTTCCCTCCAGCA |
| CDH2 | N-Cad | AGCCAACCTTAACTGAGGAGT | GGCAAGTTGATTGGAGGGATG |
| ACTC1 | Cardiac actin1 | GTCGGGACCTCACTGACTAC | CAATTTCACGTTCAGCAGTG |
| ATP2A2 | SERCA2a | TCACCTGTGAGAATTGACTGG | AGAAAGAGTGTGCAGCGGAT |
| RYR2 | RYR2 | TTGGAAGTGGACTCCAAGAAA | CGAAGACGAGATCCAGTTCC |
| BIN1 | BIN1 | ATGAGGCAAACAAGATCGCAG | CGTGACTTGATGTCGGGGAA |
| KIR2.1 | KIR2.1 | GTGCGAACCAACCGCTACA | CCAGCGAATGTCCACACAC |
| MYH6 | ɑMHC | CTCCGTGAAGGGATAACCAGG | TTCACAGTCACCGTCTTCCC |
| MYH7 | ßMHC | TCGTGCCTGA TGACAAACAGGAGT | ATACTCGGTCTCGGCAGTGACTTT |
| CACNA1c | LTCC | ACATGCTCTTCACTGGCCTC | CCCACAACAATCAAGGCGTC |
| PLN | Phospholamban | ACAGCTGCCAAGGCTACCTA | GCTTTTGACGTGCTTGTTGA |
| SLC8A1 | NCX1 | CTGGAATTCGAGCTCTCCAC | ACATCTGGAGCTCGAGGAAA |

**Supplemental Table 2.** Number of animals in each group.

|  | Week 1 | Week 4 |
| --- | --- | --- |
| Sham | 0 | 10 |
| MI | 3 | 9 |
| MI+Control-hCMP | 3 | 9 |
| MI+LBL-hCMP | 3 | 10 |
